# *Cryptococcus neoformans* Csn1201 Is Associated With Pulmonary Immune Responses and Disseminated Infection

**DOI:** 10.3389/fimmu.2022.890258

**Published:** 2022-06-02

**Authors:** Ya-li Yang, Yi-bin Fan, Lei Gao, Chao Zhang, Ju-lin Gu, Wei-hua Pan, Wei Fang

**Affiliations:** ^1^Department of Dermatology, Shanghai Ninth People’s Hospital, Shanghai JiaoTong University School of Medicine, Shanghai, China; ^2^Department of Laser and Aesthetic Medicine, Shanghai Ninth People’s Hospital, Shanghai JiaoTong University School of Medicine, Shanghai, China; ^3^Shanghai Key Laboratory of Molecular Medical Mycology, Department of Dermatology and Venereology, Changzheng Hospital, Naval Military Medical University, Shanghai, China; ^4^Department of Dermatology, Zhejiang Provincial People’s Hospital, People’s Hospital of Hangzhou Medical College, Hangzhou, China; ^5^Microscopy Core Facility, Biomedical Research Core Facilities, Westlake University, Hangzhou, China; ^6^Department of Dermatology, Third Affiliated Hospital of Naval Military Medical University, Shanghai, China

**Keywords:** *Cryptococcus neoformans*, Csn1201, immune evasion, cell wall remodelling, virulence

## Abstract

*Cryptococcus neoformans* is a major etiological agent of fungal meningoencephalitis. The outcome of cryptococcosis depends on the complex interactions between the pathogenic fungus and host immunity. The understanding of how *C. neoformans* manipulates the host immune response through its pathogenic factors remains incomplete. In this study, we defined the roles of a previously uncharacterized protein, Csn1201, in cryptococcal fitness and host immunity. Use of both inhalational and intravenous mouse models demonstrated that the *CSN1201* deletion significantly blocked the pulmonary infection and extrapulmonary dissemination of *C. neoformans*. The *in vivo* hypovirulent phenotype of the *csn1201*Δ mutant was attributed to a combination of multiple factors, including preferential dendritic cell accumulation, enhanced Th1 and Th17 immune responses, decreased intracellular survival inside macrophages, and attenuated blood–brain barrier transcytosis rather than exclusively to pathogenic fitness. The *csn1201*Δ mutant exhibited decreased tolerance to various stressors *in vitro*, along with reduced capsule production and enhanced cell wall thickness under host-relevant conditions, indicating that the *CSN1201* deletion might promote the exposure of cell wall components and thus induce a protective immune response. Taken together, our results strongly support the importance of cryptococcal Csn1201 in pulmonary immune responses and disseminated infection.

## Introduction

*Cryptococcus neoformans* is a major fungal pathogen that causes life-threatening meningoencephalitis in both immunocompromised and apparently immunocompetent hosts (1). The increasing prevalence of immunocompromised individuals, such as those with acquired immunodeficiency syndrome and organ transplantation treated with immunosuppressive compounds to prevent organ rejection, has resulted in a significant rise in the morbidity and mortality of cryptococcosis. Globally, *Cryptococcus* causes approximately 278,000 infections and 181,000 deaths each year (2). *C. neoformans* is also an excellent fungal model for studying pathogen–host interactions (3).

*C. neoformans* is widely distributed in natural environments that include soil and bird droppings (4). The desiccated yeast cells or basidiospores mainly invade the respiratory tract *via* inhalation, and then cause asymptomatic pneumonia. The pathogen is often cleared by host immunity or becomes latent (5). When host immunity is compromised or suppressed, the resting cryptococcal cells reactivate and cause systemic infection, especially in the central nervous system. Infection results from complex interactions between the pathogenic fungus and host immunity. *C. neoformans* can adapt to the hostile environment *in vivo* (such as nutrient limitation, oxidative stress, etc.) through the expression of multiple virulence factors that include capsule and melanin (6). On the other hand, the direct damage or interference caused by *C. neoformans* does not allow the host to organise an effective immune response to contain or kill the pathogen, ultimately resulting in fungal dissemination (7). Several studies have demonstrated that *C. neoformans* induces a non-protective Th2 immune response or inhibits an excessive inflammatory response through the expression of classical or non-classical virulence factors (8–13). Identification of critical pathogenic factors that control the activation of protective immune responses will be beneficial for developing new immunotherapy strategies against cryptococcosis.

Previous studies systematically screened a substantial number of uncharacterised pathogenic factors (including Csn1201) from the gene-deletion library of *C. neoformans* strain KN99α (14, 15). Their functions and mechanisms in composite cryptococcal virulence have remained unclear.

Based on the background of the clinical hypervirulent strain, H99, we further characterised the pleiotropic roles of cryptococcal Csn1201 in fungal fitness and host immunity in the present study. Deletion of Csn1201 considerably blocked the pulmonary infection and extrapulmonary dissemination of *C. neoformans*. The *in vivo* hypovirulent phenotype of the *csn1201*Δ mutant could likely be attributed to a combination of multiple factors, such as enhanced Th1 and Th17 immune responses, attenuated blood–brain barrier (BBB) transcytosis, and reduced fungal fitness in the host environment. These results enhance our understanding of host–pathogen interactions and provide opportunities to explore new intervention strategies against cryptococcosis.

## Materials and Methods

### Strains, Plasmids, and Media

*C. neoformans* strains and plasmids used in this study are listed in **Supplementary Table S1**. Strains were cultured at 30°C on YPD agar medium (1% yeast extract, 2% bacto-peptone, and 2% glucose) and selective media contained nourseothricin (100 mg/L) and/or neomycin (G418 200 mg/L). Stress media were prepared by adding different stress inducing agents to YNB agar medium (0.67% yeast nitrogen base without amino acids, 2% glucose) or YPD agar medium before autoclaving. Capsule- and melanin-inducing media were prepared as previously reported (16). DMEM with 10% FBS (Transgen Biotech, Beijing, China) was used for cell culture in the murine-derived J774 macrophage killing assay.

### Gene Disruption and Reconstitution

The *CSN1201* gene sequence (CNAG_01697) was obtained from the *C. neoformans* var. *grubii* serotype A genome database (https://www.ncbi.nlm.nih.gov/Taxonomy/Browser/wwwtax.cgi?id=235443). For gene disruption, overlap PCR was used to generate the knock-out cassette of *CSN1201*, including the flanking fragments and *NAT* resistance gene (17). The purified PCR products were precipitated onto gold microparticles and introduced into H99 cells by biolistic transformation (16). Stable transformants were screened using selective medium containing nourseothricin and confirmed by diagnostic PCR, DNA sequencing, and Southern blotting. To complement the *csn1201*Δ mutant, a genomic DNA fragment containing the ORF, promoter, and terminator region was amplified using primers ReCSN1201-F and ReCSN1201-R. This PCR fragment and the digested plasmid pJAF1 by Xba I were fused using the In-FusionH EcoDry Cloning System (Clontech; TaKaRa Bio, Shiga, Japan). The reconstructed vector pCSN1201-NEO was linearised and reintroduced into *csn1201*Δ mutants *via* biolistic transformation. Positive colonies were selected on YPD agar containing G418. Reconstitution was confirmed by diagnostic PCR and Southern blotting. The primers used to construct the strains are listed in **Supplementary Table S2**.

### Southern Blot

Southern blot analysis was performed to confirm the mutant and reconstituted strains as previously described (16). Genomic DNA (20 μg) from the strains was digested using specific restriction endonucleases and separated by 0.8% agarose gel electrophoresis. The DNA fragments were transferred to positively charged nylon membranes (Roche Applied Science, Indianapolis, IN, USA). The *NEO* or *NAT* probe was labelled with digoxigenin by the manufacturer (Roche Applied Science) and was detected on the membrane.

### *In Vitro* Phenotypic Assays

The *in vitro* stress assays were performed as previously described (17). Briefly, each strain was incubated to saturation at 30°C in YPD medium, washed, and diluted to 2.5×10^8^ cells/mL in PBS. Four microlitre aliquots were spotted onto YNB or YPD agar medium containing different stress-inducing compounds. For the oxidative and nitric oxide (NO) stress test, 2 mM hydrogen peroxide (H_2_O_2_) was added to the YNB agar medium. For the osmotic stress and high salt sensitivity tests, 1.5 M sorbitol, 1.5 M NaCl, and 1.5 M KCl were incorporated in YPD agar medium. To examine cell wall integrity, the cells were spotted onto YPD agar medium containing 0.02% SDS and 0.5% Congo red. The spotted cells were incubated at 30°C for 3 days and photographed. For temperature stress, the YPD plates were incubated at 30°C, 37°C, and 39°C.

The yeast cells were incubated in DMEM at 37°C in an atmosphere of 5% CO_2_ for 3 days in tissue culture flasks at a density of 5×10^6^ to 10^7^ cells/mL. The cells were washed with sterile PBS, and resuspended in PBS, 10 µL aliquots of the cell suspension were each mixed with a drop of India Ink and observed by microscopy. The relative ratio of the capsule was calculated as previously reported (16). For the melanisation test, cells were spotted onto caffeic acid agar and incubated for 3–7 days at 30°C. Melanin production was monitored and photographed.

The serum survival assay was performed as previously described, with modifications (18). Briefly, yeast strains were incubated overnight in YPD medium at 30°C until saturation. Cryptococcal cells were then diluted at 1:10 into mouse serum (Sigma-Aldrich, St. Louis, MO, USA), RP1640 (Sigma-Aldrich), PBS, and YPD and incubated at 37°C for another 3 days. The strains were diluted at 1:10 (1:100 overall) in PBS and plated (1:10 and 1:100) onto YPD agar plates. The plates were photographed after 3 days of incubation at 30°C.

### Transmission Electron Microscopy (TEM)

The impact of *CSN1201* deletion on the morphology of *C. neoformans* was assessed by TEM. Cryptococcal cells of both the wild-type (WT) and *csn1201*Δ strain were cultured to the logarithmic phase in YPD (30°C) or DMEM (37°C, 5% CO_2_). The cells were harvested and fixed as previously described (19). Ultrathin sections (approximately 70 nm thick) were generated using the EM UC7 ultramicrotome (Leica, Wetzlar, Germany), collected on copper grids, and stained with 2% uranyl acetate and Sato’s triple lead stain. All sections were imaged on a Talos L120C TEM (Thermo Fisher Scientific, Waltham, MA, USA) at 80 kV equipped with a 4k×4k Ceta CCD camera.

### Macrophage Killing Assay

The assay was performed as previously described (20). Yeast strains were incubated overnight at 30°C. J774A.1 macrophages seeded at a density of 10^6^ cells/well were activated with interferon-gamma (INF-γ) and lipopolysaccharide for 18 h. Prior to coincubation, the yeast cells were incubated with the monoclonal antibody mAb18B7 for 1 h. Activated macrophages were coincubated with 10^6^ cryptococcal cells for 2 h to allow phagocytosis. Extracellular yeasts were removed, diluted, and cultured on YPD plates for 3 days. Macrophages were lysed using 0.05% SDS, and intracellular (attached and digested) yeasts were determined on YPD plates after 3 days of growth. Phagocytosis efficiency was calculated as (intracellular cells/[intracellular cells + extracellular cells] × 100%). A similar method was used to calculate cryptococcal survival rates inside macrophages after 48 h of coincubation. Each strain was tested in triplicate.

### *In Vitro* BBB Model and Transcytosis Assay

An *in vitro* blood–brain barrier (BBB) model was constructed for the cryptococcal transcytosis assay as previously described (21). Briefly, 10^5^ mouse brain microvascular endothelial cells (bEND.3) were seeded into the Costar 3422 Transwell apparatus (Corning, New York, NY, USA) and grown until confluence. The integrity of the BBB model was evaluated by measuring transendothelial electrical resistance using a Millicell‐ERS apparatus (Millipore, Inc., Billerica, MA, USA). Then, 4 × 10^5^ cells of *C. neoformans* were added to the top compartment of the insert and incubated at 37°C and 5% CO_2_ for predetermined times. Cryptococci were collected from the bottom compartment (brain side) and plated onto YPD agar to determine their transmigration efficiency.

### Galleria Mellonella Virulence Assay

The yeast cells were inoculated in YPD, incubated overnight, washed, suspended and diluted to 5 × 10^6^ cells/mL in PBS. Sixteen *G. mellonella* larvae weighing 200–400 mg were inoculated for each group. The larvae suspension was injected (10 µL containing 50,000 cells) through the last foreleg and placed in petri dishes (17). The dishes were placed in a box and kept in the dark at room temperature. Survival was monitored every day. The larvae were considered dead when they displayed no movement in response to touch.

### Murine Virulence Assessment and Histological Analysis

Yeast strains were grown in YPD liquid medium at 30°C with shaking overnight. The cells were washed twice with PBS and resuspended at a final concentration of 2×10^6^ cells/mL. Groups of 10 female Balb/C mice (Shanghai Lingchuang Biotech, Shanghai, China), were infected with 25 µL (50,000 cells) yeast cells of each strain by dropping the inoculum into the nares or injecting into the tail vein. Animals were sacrificed when a loss of 20% body weight was observed compared to peak body weight. Other signs of sickness were monitored, including lethargy, lack of activity, and absence of grooming. The protocol was approved by the Ethics Committee of the Shanghai Ninth People’s Hospital

Infected animals were sacrificed at designated time points and the endpoint of the experiment. Infected lungs and brains were isolated, fixed in 4% paraformaldehyde solution, and sent to Servicebio (Shanghai, China) for processing. Tissue slides with Periodic Acid-Schiff staining were examined by microscopy. The infected lungs, spleens, and brains were obtained under aseptic conditions. The organs were weighed and macerated in 1 mL sterile PBS. Serial 10-fold dilutions of the samples were plated in duplicates 100-µL aliquots on YPD containing 100 µL/mL chloramphenicol. Colonies were counted following incubation at 30°C for 2 to 3 days. The animal protocol was approved by the Ethics Committee of the Second Military Medical University.

### Lung Leukocytes Isolation and Flow Cytometry Analysis

Lung leukocytes were isolated from inhalational mouse models as previously described (22). After sacrificing the mice, lungs were removed, minced with scissors, transferred to gentleMACS C tubes containing 5 mL of proprietary catalysts [RPMI 1640, 5% FBS, penicillin, and streptomycin; 1 mg/mL collagenase A (Roche Diagnostics); and 30 μg/mL DNase I]. The tissue samples were further homogenized using the gentle MACS dissociator (Miltenyi Biotec, Bergisch Gladbach, Germany). This process was followed by erythrocyte lysis using NH_4_Cl buffer (0.829% NH_4_Cl, 0.1% KHCO_3_, and 0.0372% Na_2_EDTA, pH 7.4). Cells were dispersed using a syringe and filtered through a sterile 100 μm nylon screen. The filtrate was centrifuged for 30 min at 1,500 g in the presence of 20% Percoll (Sigma-Aldrich) to separate leukocytes from cell debris and epithelial cells. Leukocytes were counted in a hemocytometer using Trypan blue.

Flow cytometry was performed as previously described (23). Cells were stained with antibodies (BioLegend, San Diego, CA, USA) specific to various leukocyte subpopulations according to the manufacturer’s protocols. Data were collected on a FACS LSR2 flow cytometer using FACSDiva software (Becton Dickinson Immunocytometry Systems, Mountain View, CA, USA) and analysed using FlowJo software (Tree Star, San Carlos, CA, USA). Leukocyte subpopulations were identified using the following markers: neutrophils (CD45^+^ Ly6G^+^ CD11b^+^), dendritic cells (DCs, CD45^+^ CD11c^+^ MHC II high), eosinophils (CD45^+^ CD11b^+^ Siglec F^+^), monocytes (CD45^+^ Ly6C^+^ CD11c^−^), CD4 T cells (CD45^+^ CD3^+^ CD4^+^), CD8 T cells (CD45^+^ CD3^+^ CD8^+^), and B cells (CD45^+^ CD19^+^).

### Cytokine Analysis

Cytokine analysis of lung tissues was performed as previously described (23). Briefly, lung tissue was excised and homogenized in sterile PBS at 4°C. The concentrations of each cytokine in lung homogenates were tested using the respective ELISA kit (eBioscience) according to the manufacturer’s protocol.

### Statistical Analyses

Survival was analysed using a Kaplan–Meier survival curve and the log-rank (Mantel–Cox) test. Virulence data were analysed for statistical differences with the log-rank test. A two-tailed unpaired Student *t* test was used for all other tests for statistical difference between the two groups. All statistical analyses were performed using GraphPad-Prism 5.0 software program (GraphPad, La Jolla, CA, USA). A *P*-value of < 0.05 was considered statistically significant.

## Results

### Identification and Characterisation of *CSN1201* in *C. neoformans*

A search of the *C. neoformans* genome database indicated that *CSN1201* (CNAG_01697) encodes a 472-amino acid protein containing a PCI domain (Proteasome, COP9, Initiation factor 3). Bioinformatics analysis unexpectedly revealed the putative Csn1201 orthologue only in *Cryptococcus* spp. and evolutionarily related basidiomycete (such as *Kwoniella* spp., *Saitozyma* spp., and *Trichosporon* spp.). The orthologue was not present in *Saccharomyces cerevisiae* and higher eukaryotic species (such as *Caenorhabditis elegans*, *Drosophila melanogaster*, and *Homo sapiens*). The findings suggest that Csn1201 might be evolutionarily specific to some fungal species, including *C. neoformans*.

### Roles of Csn1201 in Stress Responses, Capsule Production, and Melanin Secretion of *C. neoformans*

**Figure 1:**
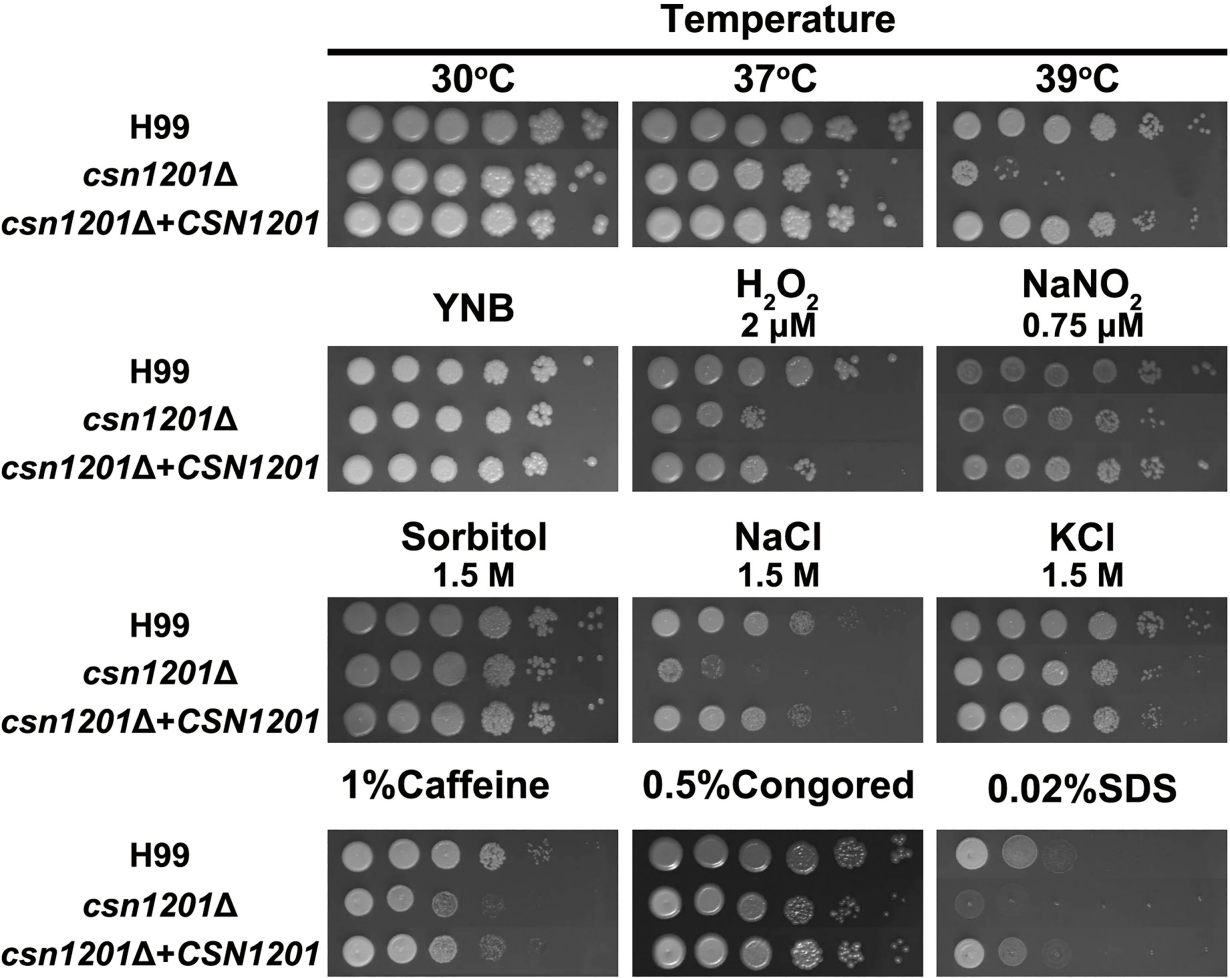
Csn1201 is involved in various stress responses of *C. neoformans*. Strains were grown to saturation at 30°C in YPD medium, washed, serially diluted 10-fold (1–10^6^ dilutions), and spotted (4 µL) onto YNB or YPD agar medium containing different stress-inducing agents. Spotted cells were incubated at 30°C for 3 days and then photographed.

To investigate the roles of Csn1201 in the growth and pathogenesis of *C. neoformans*, we constructed the mutant strain *csn1201*Δ and its reconstituted strain *csn1201*Δ+*CSN1201 via* biolistic transformation. We first tested the sensitivity of each strain to a variety of *in vitro* stresses. As shown in **Figure 1**, the *csn1201*Δ mutant exhibited temperature sensitivity with a partial growth defect at 37°C and 39°C. Furthermore, *csn1201Δ* showed increased sensitivity to 2 µM H_2_O_2_ and 1.5 M NaCl, compared with the WT strain. These findings suggested that *CSN1201* might be involved in regulating cryptococcal tolerance to oxidative stress and high salt environments. The *csn1201*Δ mutant also displayed mild defects in cryptococcal tolerance to cell wall or membrane-damaging agents. The *csn1201*Δ+*CSN1201* reconstituted strain completely restored WT sensitivity in the *in vitro* stress assays.

**Figure 2:**
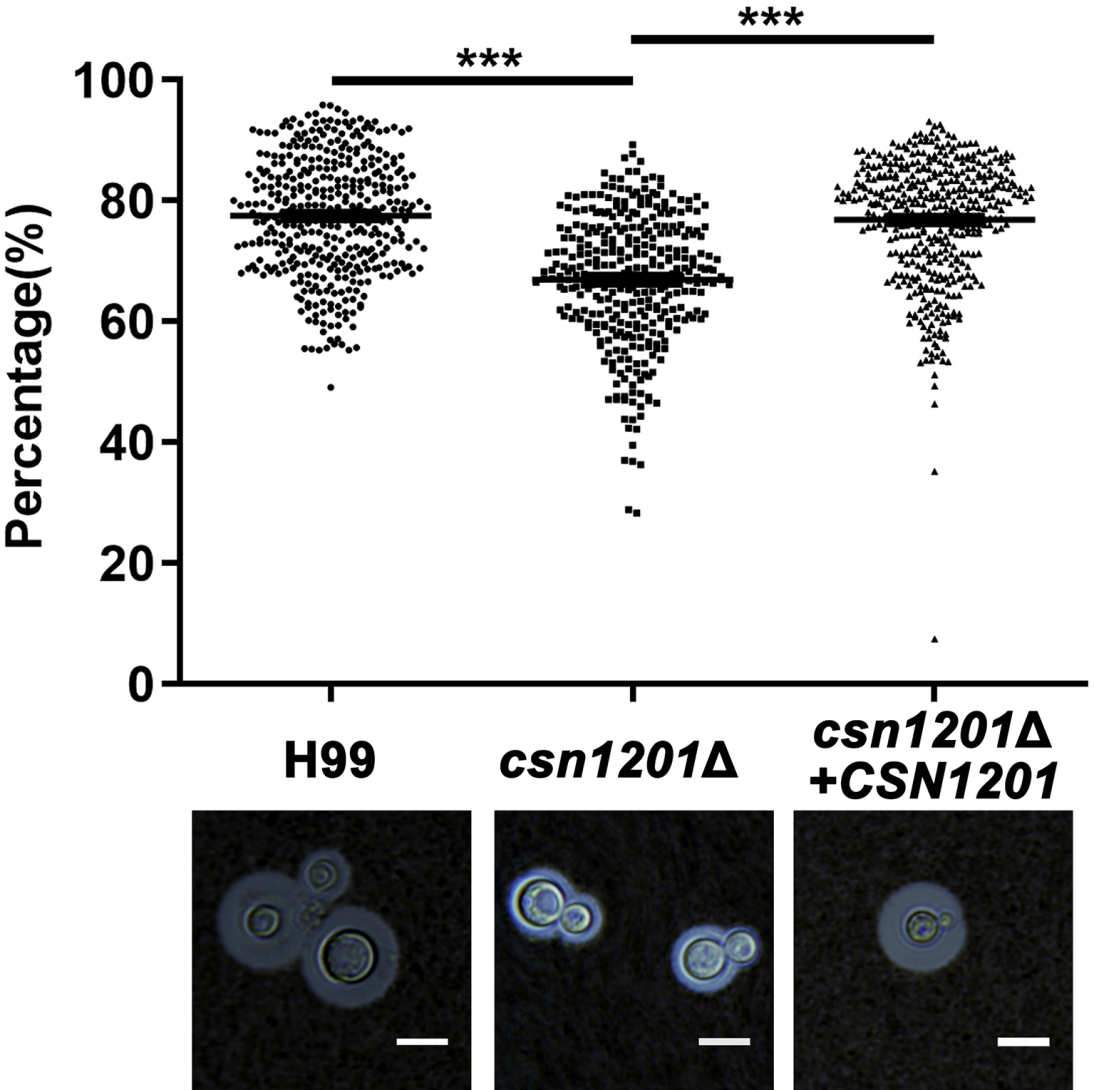
The *CSN1201* deletion down-regulates capsule production. Each strain was cultured on DME medium for capsule production at 37°C in a 5% CO_2_ atmosphere for 3 days. The capsule was detected by staining with India ink and visualising at 100× magnification (scale bar = 10 µm). Total (cell and capsule) and cell-only diameters were measured using Photoshop Software for at least 300 cells for each strain. ***, *P* < 0.001.

We next evaluated the effect of *CSN1201* deletion in the production of different pathogenic factors of *C. neoformans*. When incubated in DMEM, the capsule thickness of the mutant was significantly decreased. The capsule thickness was restored to that of the WT in the reconstituted strain (**Figure 2**). When grown in melanin-inducing medium containing caffeic acid or niger seed, no noticeable melanin changes were observed in these strains (**Figure S1**). These results implicate Csn1201 in cryptococcal capsule production but suggest that Csn1201 is not required for melanin accumulation.

### Attenuation of Cryptococcal Survival in Macrophages by *CSN1201* Deletion and Virulence *In Vivo* in a *G. mellonella* Model

**Figure 3:**
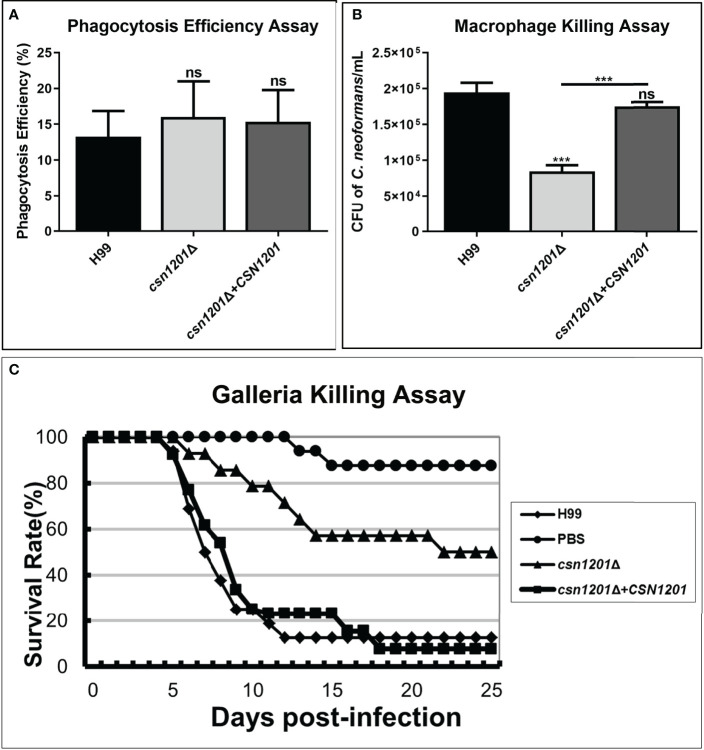
Csn1201 is required for cryptococcal survival in macrophages and in *Galleria mellonella*. Macrophage phagocytosis **(A)** and killing **(B)** assays. Activated macrophages were coincubated with 10^6^ cryptococcal cells for 2 h to allow phagocytosis. Extracellular yeasts were removed, diluted, and cultured on YPD plates for 3 days. Macrophages were lysed with 0.05% SDS and intracellular (attached and digested) yeasts were determined on YPD plates after 3 days of growth. Phagocytosis efficiency was calculated using the formula (intracellular cells/[intracellular cells + extracellular cells] × 100%). A similar method was used to calculate cryptococcal survival inside the macrophage after 24 h of coincubation. Each strain was tested in triplicate. ***, *P* < 0.001; ns, no significant difference. **(C)** *In vivo* virulence assay in the *G. mellonella* model.

Macrophages, as the first line of host defence, are critical for cryptococcal latency and dissemination (24). Thus, we developed an *ex vivo* cell model to examine the intracellular parasitic capacity of the *csn1201Δ* mutant strain. There was no significant difference in the phagocytosis efficiency of the macrophages for these strains (**Figure 3A**), indicating that the *CSN1201* deletion did not affect cryptococcal cell uptake into macrophages. After 48 h of co-culture with activated J774.1 macrophage-like cells, the mutant strain exhibited approximately a 50% reduction in intracellular survival (*P<*0.001) within macrophages compared with that of the WT or reconstituted strain (**Figure 3B**).

To further exclude the potential effect of high-temperature sensitivity on cryptococcal virulence, we also constructed the *G. mellonella* non-vertebrate infection model at 25°C. In the *G. mellonellar*/*C. neoformans* system, the *csn1201Δ* mutant (LT_50_ = 22 days) exhibited significantly attenuated virulence compared with the WT (LT_50_ = 7 days) or reconstituted (LT_50_ = 9 days) strains, as determined by survival analysis (*P<*0.01, **Figure 3C**). These findings indicate that the *CSN1201* deletion might attenuate the virulence of *C. neoformans* independently of its influence on thermotolerance.

### Attenuation of *C. neoformans* Virulence by *CSN1201* Deletion in an Inhalational Mouse Model

**Figure 4:**
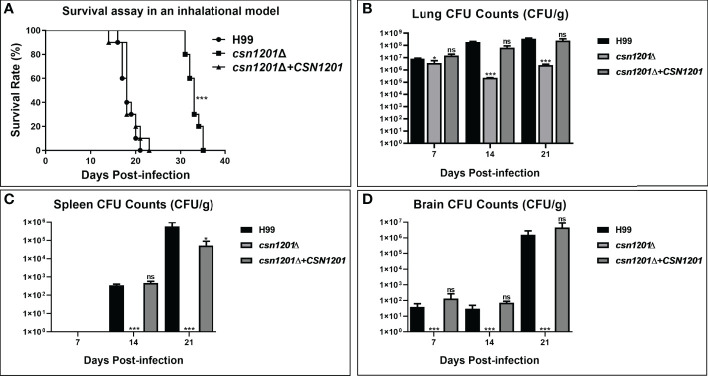
Csn1201 is essential for *C. neoformans* virulence in mouse inhalational infection models. **(A)** Survival assay in an inhalational model. Mouse lung **(B)**, spleen **(C)**, and brain **(D)** fungal burden assays were performed by counting fungal colonies after culturing serially diluted tissue lysate on YPD plates. *, *P* < 0.05; ***, *P* < 0.001; ns, no significant difference.

We further assessed the virulence of the *csn1201Δ* mutant in a murine inhalation model (**Figure 4**). Briefly, 10 female Balb/c mice were intranasally inoculated with 10^5^ C*. neoformans* cells for each strain. Mice were monitored daily and sacrificed at predetermined clinical endpoints. All mice infected with the WT or reconstituted strains died within 23 days. Their average survival time was 18.3 ± 1.64 days and 18.6 ± 2.37 days, respectively. However, all mice infected with the *csn1201Δ* mutant succumbed to the infection by day 35. Their average survival time was extended to 32.9 days (*P*<0.001), suggesting a significant virulence defect (**Figure 4A**). Fungal burdens in different organs of each group were also examined at different time points. Viable yeast cells were discovered from the lungs of mice infected with the *csn1201Δ* mutant, but not from the spleen and brain. Intriguingly, a transient reduction in yeast viable numbers (2.28×10^5^ ± 7.2×10^3^ colony forming units (CFU)/g) was observed in the lungs of the *csn1201Δ* mutant group by 14 days, which was increased (2.47×10^6^ ± 4.6×10^5^ CFU/g) by 21 days (**Figures 4B–D**). In contrast, WT or reconstituted strain infected mice displayed significantly higher fungal burdens in all the tested organs at each time point (*P*<0.05). These results suggest that Csn1201 is essential for the pathogenesis of *C. neoformans* and is especially required for hematogenous dissemination from a pulmonary infection.

### Defects of *Csn1201Δ* Mutant in Serum Survival and BBB Transcytosis *In Vitro*

**Figure 5:**
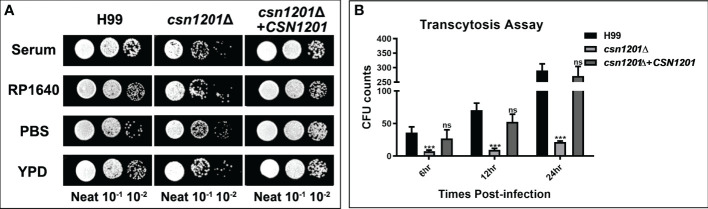
The *CSN1201* deletion is involved in serum survival and *in vitro* blood-brain barrier transcytosis of *C. neoformans.* **(A)**. Serum survival assay. Each strain was grown to saturation in YPD at 30°C, diluted 1:10 in mouse serum or YPD, and incubated at 37°C for 4 days. “Neat” represents the colony-forming units (CFU) concentration in serum. Strains were serially diluted 10-fold prior to being spotted onto YPD plates. **(B)**. In vitro transcytosis assay of *C. neoformans* at 6, 12, and 24 hr. ***, *P* < 0.001; ns, no significant difference.

*C. neoformans* is a facultative intracellular pathogen that can evade the phagocytic cells in the extracellular environment (25). To determine the reason underlying the defective extrapulmonary dissemination by the *csn1201Δ* mutant, we assessed its ability to survive in serum. When exposed to human serum at 37°C for 4 days, the mutant strain displayed mild growth inhibition (**Figure 5A**). The survival defect observed in serum was not associated with nutrient starvation but contributed to enhanced high-temperature susceptibility, as the *csn1201Δ* mutant exhibited similar survival in RP1640, YPD medium, and PBS. We also tested the effects of *CSN1201* deletion on cryptococcal transmigration across host brain microvascular endothelial cells *via* an *in vitro* BBB system. A significant reduction (86.5% at 12 h and 92.5% at 24 h) was noted in the transcytosis efficiency of the *csn1201Δ* mutant. The transmigration rate returned to that of the WT in the reconstituted strain (**Figure 5B**). Together, these data indicate that Csn1201 might promote the serum survival and BBB transcytosis of *C. neoformans*.

### Attenuated Virulence, but Distinct Pathogenic Pattern, of the *Csn1201Δ* Mutant in the Intravenous Mouse Model

**Figure 6:**
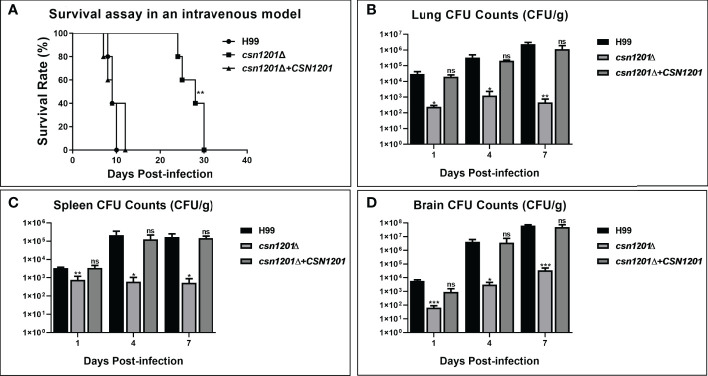
The *CSN1201* deletion attenuates virulence of *C. neoformans* in a murine intravenous model. **(A)** Survival assay in mouse intravenous cryptococcosis with different strains. Fungal burden assays in the lung **(B)**, spleen **(C)**, and brain **(D)**. Organs were removed at 1, 4, and 7 days post-infection in the three groups.*, *P* < 0.05; **, *P* < 0.01; ***, *P* < 0.001; ns, no significant difference.

To further dissect the roles of Csn1201 in cryptococcal virulence and extrapulmonary dissemination, an intravenous mouse model was constructed as previously described. As expected, the *CSN1201* deletion significantly prolonged the survival of mice intravenously infected with *C. neoformans* (27.4 ± 2.79 days, *P<*0.01 compared with the WT). In contrast, the average survival times of WT and *csn1201Δ+CSN1201* mice were 9.2 ± 0.84 and 9.6 ± 2.30 days, respectively (**Figure 6A**). The fungal burden assay revealed a drastic reduction of fungal burden in different organs (lungs, spleens, and brains) from *csn1201Δ*-infected mice at each time point (1, 4, and 7 days post-infection; *P<*0.05, compared with the WT). However, distinct from the inhalational model (**Figure 4**) the *csn1201Δ* mutant still invaded the spleen and brain despite a remarkably lower fungal burden (**Figures 6B–D**). These results indicate that Csn1201 is required for the disseminated infection of *C. neoformans* but plays different roles in different infection routes.

### Altered Pulmonary Inflammation and Immune Responses to the *Csn1201*Δ Mutant in the Inhalational Mouse Model

**Figure 7:**
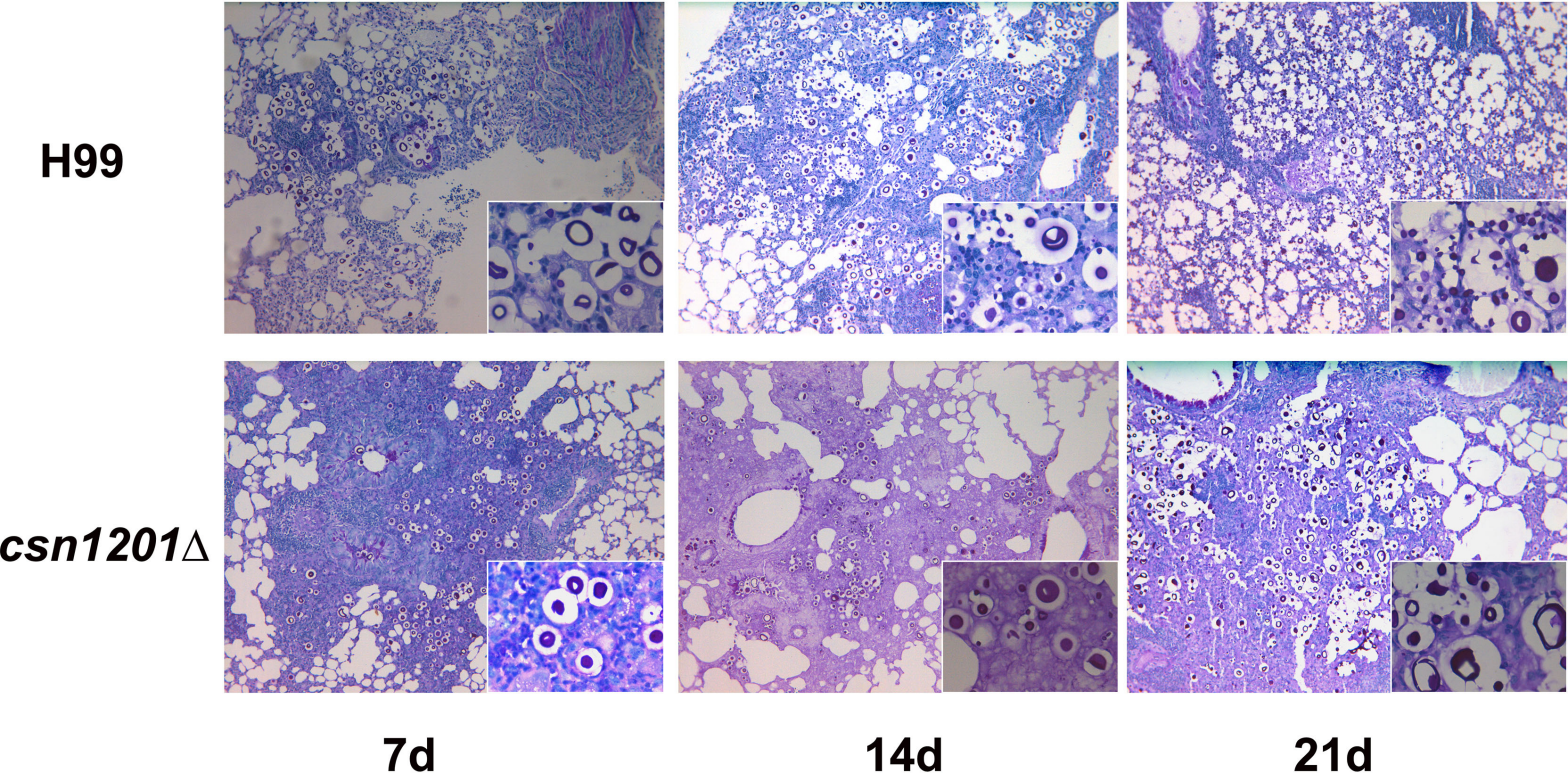
Csn1201 regulates host pulmonary inflammatory responses to *C. neoformans*. Representative photographs of haematoxylin and eosin-stained mouse lungs at different times post-infection.

Based on the above results, we speculated that the absence of the *csn1201Δ* mutant strain in extrapulmonary dissemination might be attributed to its inability to breach the pulmonary immune defence in the inhalational model. To define the role of Csn1201 in pulmonary pathogenesis, we performed histological analyses of infected lungs at various time points in BALB/c mice (**Figure 7**). In BALB/c mice infected with the WT strain, a few yeast cells were confined to the alveolar space or the septum along with mild infiltration by neutrophils at 7 days post-infection. However, on day 14 post-infection, some of the alveolar architecture was destroyed because of the propagated cryptococci and severe inflammation. The inflammatory cells primarily comprised mononuclear cells with a few eosinophils. Pulmonary alveoli displayed diffuse destruction and were filled with a full field of fungal cells, but much fewer inflammatory cells at 21 days post-infection. Consistent with the quantitative fungal burden assay, the *csn1201Δ* strain showed attenuated proliferation in the lungs of mice. Yeast cells were primarily localized in interstitial tissues and were surrounded by marginal zones of mixed leukocyte infiltrates that formed diffuse granulomas at 7 days post-infection. The inflammatory cells primarily comprised epithelioid cells mixed with neutrophils and lymphocytes. Similarly, fungal cells were confined to pulmonary interstitia with reduced inflammation at 14 days post-infection. However, on day 21 post-infection, expansive cryptococcal clusters of the mutant were released from damaged epithelioid cells and caused severe destruction of lung tissues.

**Figure 8:**
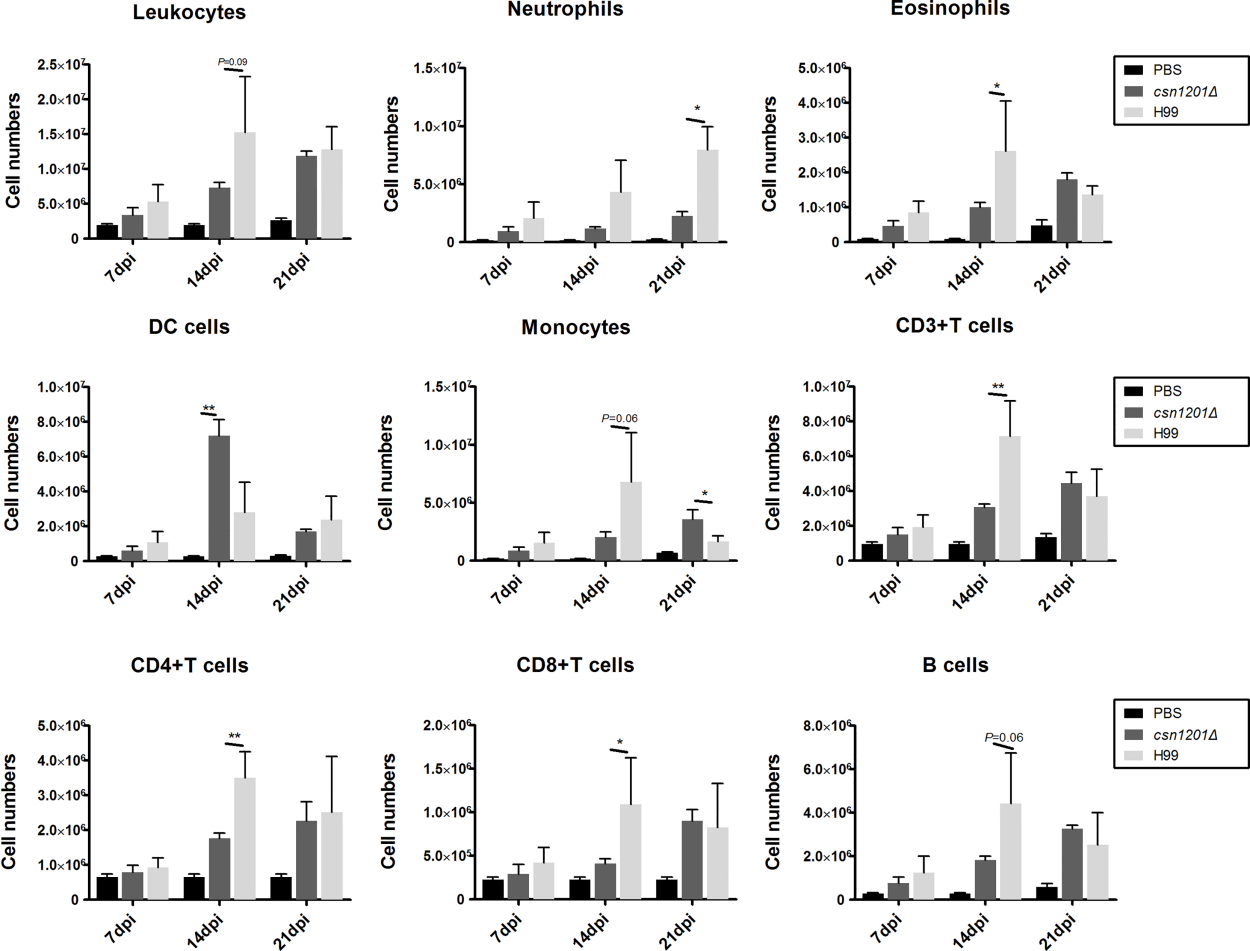
The *CSN1201* deletion alters the pulmonary inflammatory recruitment pattern during cryptococcal infection. Subsets of pulmonary immune cells from mice infected with *C. neoformans* were assessed by flow cytometry. Values are the mean ± SEM, n = 4 or more mice per group. *, *P* < 0.05; **, *P* < 0.01.

**Figure 9:**
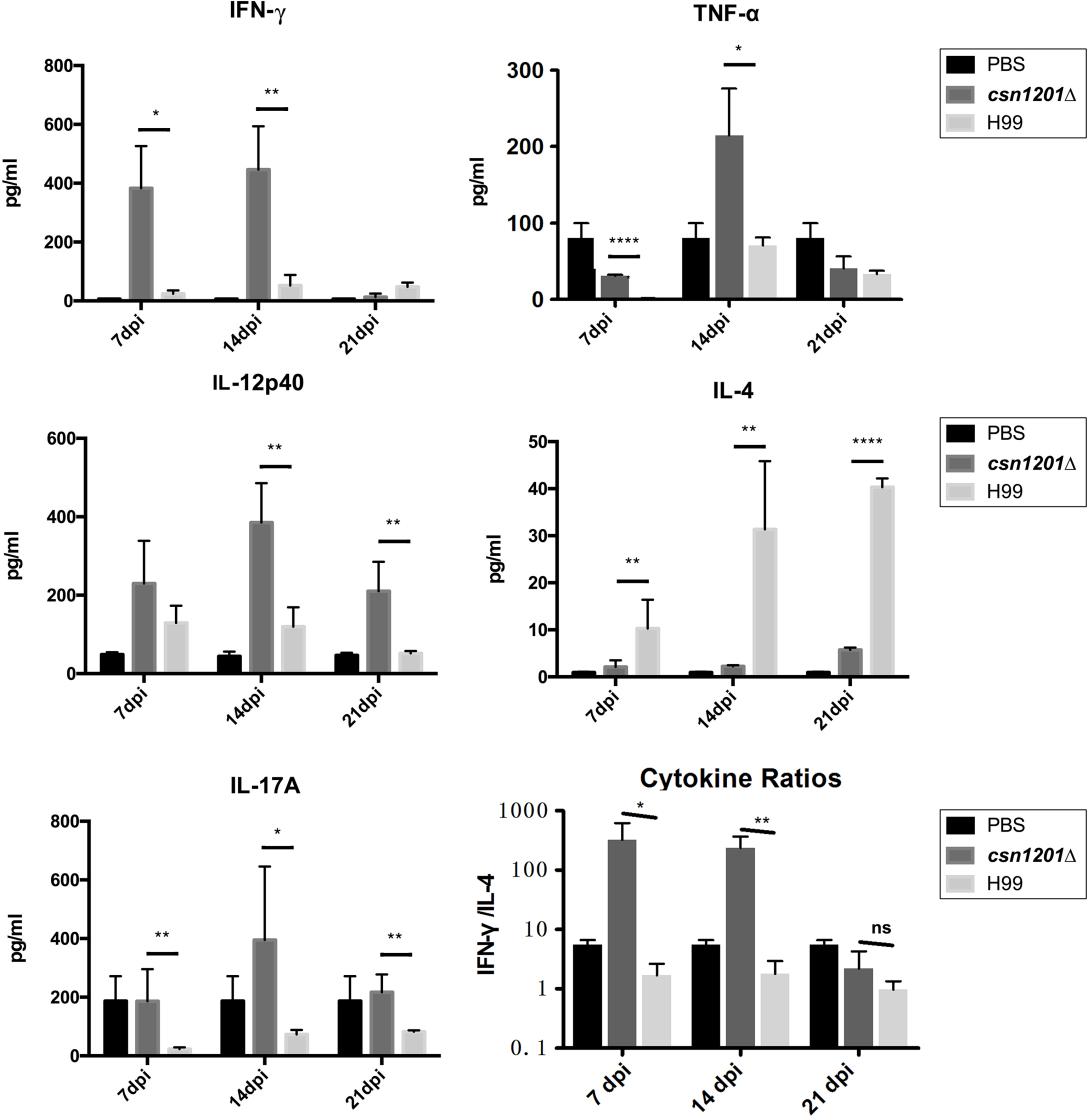
The *CSN1201* deletion promotes robust development of pulmonary Th1 and Th17 cytokine bias during cryptococcal infection. Specified inflammatory cytokines of *C. neoformans-*infected mice were analysed using ELISA. The Th1/Th2 bias ratio was calculated as IFN−γ/IL−4. Values are the mean ± SEM, n = 4 or more mice per group. *, *P* < 0.05; **, *P* < 0.01; ****, *P* < 0.0001; ns, no significant difference.

To assess the effect of cryptococcal Cns1201 deletion on pulmonary cellular responses, we isolated leukocytes from the lungs and conducted flow cytometry analysis at different time points (**Figure 8**). On day 7 post-infection, no significant difference was observed in the absolute numbers of total leukocytes and each subpopulation isolated from different groups. On day 14 post-infection, mouse lungs infected with the *csn1201Δ* strain showed substantially fewer total leukocytes, monocytes, T cells (including both CD4 + T cells and CD8 + T cells), B cells, and eosinophils, but significantly more dendritic cells than in the WT-infected mouse lungs. The lungs of *csn1201Δ* infected mice harboured fewer neutrophils, but significantly more monocytes, than those in the WT-infected group 21 days post-infection. Accordingly, the *CSN1201* deletion induced a different pattern of pulmonary cytokines in *C. neoformans*-infected mice (**Figure 9**). The levels of IFN-γ, tumour necrosis factor-alpha (TNF-α), interleukin (IL)-12, and IL-17 in the *csn1201Δ*-infected mice were profoundly higher than those in the WT-infected mice, whereas expression of IL-4 was substantially diminished in the *csn1201Δ*-infected group compared with that in the WT-infected group. Thus, the *CSN1201* deletion in *C. neoformans* altered the pulmonary inflammation pattern and promoted T cell polarization towards the protective Th1 and Th17 phenotypes.

### Involvement of Csn1201 in Cell Wall Remodelling of *C. neoformans* Under Host-Relevant Conditions

**Figure 10:**
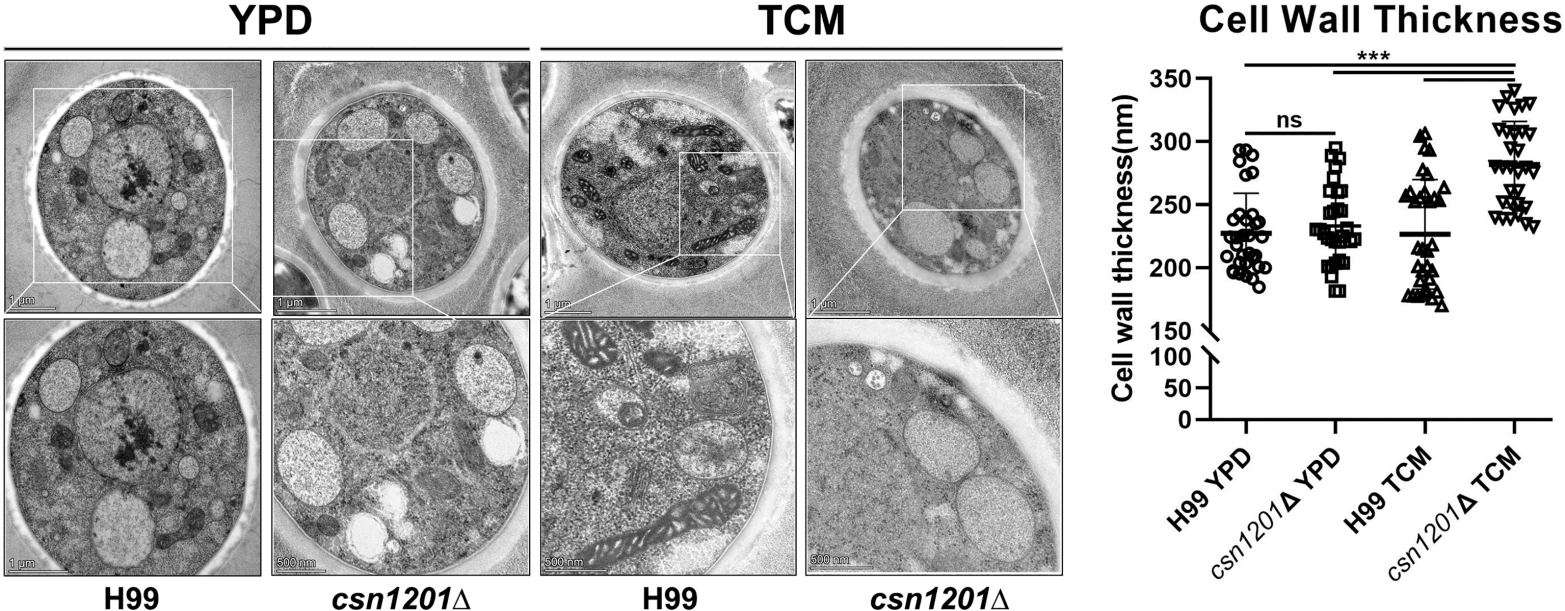
Csn1201 regulates cell wall thickness of *C. neoformans* under host-relevant conditions. Transmission electron microscopy of both the WT and *csn1201*Δ strains was carried out after incubation in either YPD medium or DMEM. ImageJ was utilized to quantify cell wall thickness. Results represent the mean ± SEM. ***, *P* < 0.001; ns, no significant difference.

Alteration of pulmonary inflammation and immune responses observed in the *csn1201*Δ mutant infection suggest that the *CSN1201* deletion might aberrantly expose an antigenic trigger of *C. neoformans* under the host environment *in vivo*. To test this hypothesis, we examined the effects of *CSN1201* deletion on cryptococcal cell morphology, especially on the cell wall, using TEM. In YPD medium, there was no significant difference in cell morphology and cell wall thickness between the WT and mutant strains (H99, 227.4 ± 31.79 nm; *csn1201Δ*, 233.3 ± 30.87 nm). However, under tissue culture conditions (DMEM with 5% CO_2_ at 37°C), the mutant cells showed a remarkable increase in cell wall thickness compared to the WT cells (H99, 226.6 ± 43.37 nm; *csn1201Δ*, 281.9 ± 34.22 nm; *P<*0.001) (**Figure 10**). Additionally, we observed a noticeable increase in intracellular vacuoles and their matrixes in the mutant cells. These results indicate that Csn1201 might be implicated in the cell wall remodelling of *C. neoformans* under host-relevant conditions.

## Discussion

In this study, we defined the roles of the uncharacterised protein, Csn1201, in cryptococcal virulence during pulmonary and disseminated infection. Deletion of *CSN1201* significantly weakened the survival of *C. neoformans* within the lungs of infected mice. The decreased microbial burden was closely associated with protective immune responses, which were probably provoked by the exposure of cell wall components in the *csn1201Δ* mutant. Furthermore, *CSN1201* expression also promotes the disseminated infection of *C. neoformans*. The decreased capacity of the *csn1201Δ* mutant for extrapulmonary dissemination was probably attributed to a combination of multiple factors, such as reduced fungal fitness under host conditions, attenuated BBB transcytosis, and pulmonary immune response. Overall, this study significantly advances the understanding regarding the interaction of cryptococcal pathogenic factors with host defences *in vivo*.

Previous studies identified Csn1201 as an uncharacterised virulence regulator of *C. neoformans* based on a large-scale screening of cryptococcal mutant libraries (14, 15). Herein we focussed on the effects of Csn1201 on host defence against a hypervirulent *C. neoformans* strain, H99, in multiple animal models. Results from murine models showed significantly prolonged survival and accelerated fungal clearance from different organs in *csn1201Δ*-infected mice, indicating that Csn1201 is required for pulmonary invasion and extrapulmonary dissemination of *C. neoformans*. Several lines of evidence indicate that the regulatory role of Csn1201 in virulence appears to be linked with fungal immune responses, rather than exclusively with the pathogenic fitness of *C. neoformans*. First, the *csn1201Δ* mutant displayed relatively good (although diminished) growth in mouse serum at 37°C, despite the mild defect in thermotolerance. Secondly, the *CSN1201* deletion significantly attenuated cryptococcal virulence in the *G. mellonella* model at room temperature. *G. mellonella* is a facile invertebrate host to study fungal pathogenesis, which has an innate immune system similar to that of mammalians (26). Our data indicate that Csn1201 expression contributes to cryptococcal evasion of host innate immune responses, which is independent of its influence on fungal thermotolerance. Most importantly, viable yeast cells could be isolated from the spleen and brain of mice intravenously infected with the *csn1201Δ* mutant, but not in the inhalational model, indicating that the pulmonary immune response provoked by the mutant could block its extrapulmonary dissemination during natural disease progression.

The immunomodulatory role of cryptococcal Csn1201 was emphasized by pulmonary immunopathological analysis in the inhalational mouse model. The lungs of mice infected with *csn1201Δ* were characterised by granuloma formation in the early and middle stages of infection. *Cryptococci* were contained in interstitial tissues and surrounded by mixed inflammatory infiltrates, suggesting that the host immune system recognized and attempted to limit the *csn1201Δ* infection. Consistently, the *CSN1201* deletion induced remarkable alleviation of cryptococcal expansion and overall inflammatory infiltration (typically by 14 days post-infection) in contrast to the uncontrolled growth and rapid extrapulmonary dissemination of the WT strain. Despite a weaker inflammatory response, infection with the *csn1201Δ* mutant induced recruitment of more dendritic cells at 14 days post-infection in the lungs. As critical innate immune cells, DCs can phagocytose and kill invading pathogens, as well as direct the adaptive immune response *via* antigen presentation (27, 28). The specific cytokine milieu (such as IFN-γ) or cell wall component exposure (such as mannoprotein) could stimulate protective immunity against *C. neoformans* by inducing early recruitment and activation of dendritic cells (29–31). We speculate that the *csn1201Δ* mutant might exploit a similar mechanism to stimulate the protective immunity of dendritic cells during the innate phases. This suggestion should be assessed. Furthermore, infection with the *csn1201Δ* mutant also resulted in a significant reduction of eosinophil and neutrophil infiltration during the middle and late phase. Eosinophils are a major source of IL-4 and contribute to a non-protective Th2 immune response, especially allergic inflammation during pulmonary cryptococcosis (32). Neutrophil depletion induces protective anti-fungal immunity by increasing inflammatory cytokine production and decreasing off-target tissue damage in the lungs (33, 34).

Cytokine analyses of pulmonary homogenates provided additional robust evidence of the effects of Csn1201-mediated, non-protective, immune modulation. On one hand, cryptococcal Csn1201 significantly enhanced the expression of Th2 cytokine IL-4 in the lungs of H99-infected mice, consistent with the accumulation of lung eosinophils. On the other hand, the *CSN1201* deletion induced significant upregulation of Th1 cytokines (IFN-γ and IL-12) and inflammatory cytokines (TNF-α and IL-17). These upregulated cytokines are essential for protective immunity against *C. neoformans* infection (22, 35–37). Of the aforementioned cytokines, IFN-γ is probably crucial for the *csn1201Δ*-induced protective immune responses. Previous studies have reported that infection with an IFN-γ-producing strain of *C. neoformans* induces similar alteration of cytokine profiles in mouse lungs as observed in the present study, but this is not observed in mice infected with a TNF-α-producing strain (22, 31, 38).

The protective immune response is probably attributed to the exposure of cell wall components in the *csn1201Δ* mutant. We observed that the *CSN1201* deletion noticeably enhanced cell wall thickness and mildly decreased extracellular capsule thickness under host-relevant conditions in *C. neoformans*. In addition, the *csn1201Δ* mutant exhibited enhanced sensitivity to cell wall or membrane-damaging agents. Alteration of cell wall components or its regulators in *C. neoformans* reportedly stimulate aberrant host immune responses and thus change the outcomes of cryptococcal infection (12, 39, 40). A prior bioinformatics analysis suggested that the PCI domain mediates and stabilises protein–protein interactions within multiprotein complexes, such as the 26S proteasome lid and the COP9 signalosome (41). Therefore, we speculate that Csn1201 might exploit a similar mechanism to regulate cell wall remodelling and thus alter the host immune response. This remains to be investigated.

In addition to modulating pulmonary immunity, Csn1201 is also essential for the extrapulmonary dissemination of *C. neoformans*. Regardless of the infection route, the *csn1201Δ* mutant exhibited a significantly reduced infectivity in different murine organs (especially the central nervous system). The decreased pathogenicity of *csn1201Δ* was closely associated with reduced fungal fitness under stressful host conditions *in vivo*. The *CSN1201* deletion significantly weakened cryptococcal tolerance to various stressors *in vitro*, intracellular survival in macrophages, and transcytosis efficiency across the BBB. *C. neoformans* utilizes multiple virulence factors to adapt to the hostile environment *in vivo* (42). Csn1201 positively regulates melanin production but has no effect on capsule synthesis in the WT strain, KN99α (14, 15). However, in the present study, the *CSN1201* deletion in the H99 hypervirulent strain resulted in reduced capsule size and a mild growth defect at the host temperature, but did not affect melanisation, which reflects a functional reconfiguring of homologous genes during cryptococcal microevolution. Each pathogenic factor provides a relatively distinct contribution to the overall virulence of *C. neoformans*. The regulatory role of Csn1201 in cryptococcal pathogenicity is dependent on a combination of multiple pathogenic factors and their interaction with the host. More importantly, Csn1201 might be a unique protein that is evolutionarily specific to some fungal species, facilitating the future development of therapeutic interventions against cryptococcosis.

In summary, our study provides novel insights into the role of the cryptococcal Csn1201 protein in a hypervirulent *C. neoformans* strain. Csn1201 promotes pulmonary growth and extrapulmonary dissemination by modulating both innate and adaptive host responses alongside fungal pathogenic fitness. Further experiments are necessary to determine how *C. neoformans* utilizes Csn1201 to regulate the molecules on the surface of pathogens that can avoid host immune activation, thus leading to cryptococcal pathogenesis.

## Data Availability Statement

Publicly available datasets were analyzed in this study. This data can be found here: https://www.ncbi.nlm.nih.gov/Taxonomy/Browser/wwwtax.cgi?id=235443.

## Ethics Statement

The animal study was reviewed and approved by the Ethics Committee of the Shanghai Ninth People’s Hospital.

## Author Contributions

Y-lY, Y-bF, J-lG, and WF conceived, designed and performed the experiments. LG, CZ, and WF analysed the data. J-lG, W-hP, and WF drafted the manuscript. All authors contributed to the article and approved the submitted version.

## Funding

This study was supported by the National Science Foundation of China (81501728, 82072259, 81772159, 82073453 and 81902041).

## Conflict of Interest

The authors declare that the research was conducted in the absence of any commercial or financial relationships that could be construed as a potential conflict of interest.

## Publisher’s Note

All claims expressed in this article are solely those of the authors and do not necessarily represent those of their affiliated organizations, or those of the publisher, the editors and the reviewers. Any product that may be evaluated in this article, or claim that may be made by its manufacturer, is not guaranteed or endorsed by the publisher.

## Supplementary Material

Supplementary Figure 1 Melanin production assay. Strains were grown on Niger Seed or Caffeic Acid medium at 30°C.

Supplementary Table 1 Strains and plasmids used in this study.

Supplementary Table 2 Primers used in this study.
