# Supplementary material for: Cryptococcus neoformans Csn1201 Is Associated With Pulmonary Immune Responses and Disseminated Infection
Source: Front Immunol. 2022 Jun 2;13:890258. doi: 10.3389/fimmu.2022.890258 (PMC9201341; doi:10.3389/fimmu.2022.890258)
Supplement: Supplementary Figure 1 — Melanin production assay. Strains were grown on Niger Seed or Caffeic Acid medium at 30°C. [file DataSheet_1.docx]

**Supplemental Materials**

**Figure S1. Melanin production assay.** Strains were grown on Niger Seed or Caffeic Acid medium at 30°C.

**
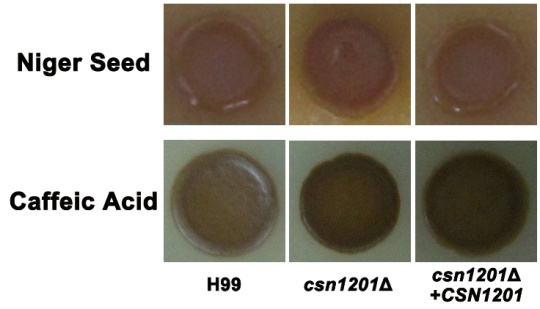
**

**Table S1. Strains and plasmids used in this study.**

| Strain & Plasmid | Genotype | Reference |
| --- | --- | --- |
| ***C. neoformans*** |  |  |
| H99 | *MATα* | Perfect et al. (1993) |
| *csn1201*Δ | *MATαcsn1201::NAT* | This study |
| *csn1201*Δ*+CSN1201* | *MATαcsn1201::NAT+CSN1201::NEO* | This study |
|  |  |  |
| **Plasmid** |  |  |
| pJAF1 | *NEO* resistance gene | O'Meara TR et al.(2010) |
| pCH233 | *NAT* resistance gene | O'Meara TR et al.(2010) |
| pCSN1201-NEO | Modified pJAF1 containing gene *CSN1201* | This study |
| pFLAG-CSN1201 | Modified pJAF1 containing *FLAG* fragment and gene *CSN1201* | This study |

**Table S2. Primers used in this study.**

| Primer | Sequence(5’-3’) | Function |
| --- | --- | --- |
| CSN1201-5UTR-F | CTCTGTCAAGGAGCGATATT | Construction of knocking-out cassatte |
| CSN1201-5UTR-R | GTCATAGCTGTTTCCTGTGAACTGAAAGAAGGAGAGG | Construction of knocking-out cassatte |
| CSN1201-3UTR-F | CTGGCCGTCGTTTTACTACCAGGGTTTGATACTTGG | Construction of knocking-out cassatte |
| CSN1201-3UTR-R | TCAGATCCCTTACGTCATTC | Construction of knocking-out cassatte |
| CSN1201-DP-F | CTAGTACAATTCCTGGGACG | Analysis of proper homologous recombination of *csn1201∆* |
| ReCSN1201-F | GCTCTAGACTCTGTCAAGGAGCGATATT | Reconstitiution for *csn1201∆* mutant |
| ReCSN1201-R | GCTCTAGATCAGATCCCTTACGTCATTC | Reconstitiution for *csn1201∆* mutant |
| ReCSN1201-DP-R | TTAAGCGTCGAGTGGAGGA | Analysis of reconstituted strains (*csn1201*Δ*+CSN1201*) |
| M13F | GTAAAACGACGGCCAG | Construction of knocking-out cassatte |
| M13R | CAGGAAACAGCTATGAC | Construction of knocking-out cassatte |
| NEO-F | TATGTCCTGATAGCGGTCCG | Creation of NEO Probe for Soutern analysis |
| NEO-R | AAGATGGATTGCACGCAGG | Creation of NEO Probe for Soutern analysis |
| Nat-F | ACCTCTGGCTGGAGGTCAC | Creation of NAT Probe for Soutern analysis |
| Nat-R | GGGCATGCTCATGTAGAGC | Creation of NAT Probe for Soutern analysis |
